# Editorial: Use of DCE-MRI in female affecting cancers

**DOI:** 10.3389/fonc.2023.1260469

**Published:** 2023-08-11

**Authors:** Valeria Romeo, Carlo Cavaliere

**Affiliations:** ^1^ Department of Advanced Biomedical Sciences, University of Naples “Federico II”, Naples, Italy; ^2^ IRCCS Synlab SDN, Naples, Italy

**Keywords:** breast cancer, ovarian cancer, artificial intelligence, radiomics, machine learning, magnetic resonance imaging

Medical pictures are employed as a primary evaluation and measuring tool for the screening, diagnosis, staging, and direction of cancer therapy, having undergone a significant evolution in the function they play in cancer care from complementary information. The introduction of new imaging probes and contrast agents, as well as improvements in image processing and analysis methods, have all contributed to its evolving position in the therapy of cancer. This includes cutting-edge imaging methods that probe features of the physiology and microstructure of tissues as well as the biology of tumors, such as tumor metabolism, perfusion, and oxygenation (1).

The medical imaging field has incorporated imaging techniques to correlate the pathological and molecular characteristics of a tumor as well as to measure early changes in tumors that may serve as useful biomarkers of therapy response or tumor progression to support the goals of pursuing personalized, accurate, and evidence-based cancer care (2).

One of the most recent imaging methods, dynamic contrast-enhanced magnetic resonance imaging (DCE-MRI), is frequently used to diagnose, describe, and provide prognoses for individuals with cancer as well as other disorders. The ability to distinguish between benign and malignant tumors, provide greater clarity in tissue characterization, enable differential diagnoses, and provide insight into vascularization, perfusion, and capillary permeability, which can be very useful in predicting metastasis to secondary sites through the process of angiogenesis, are all advantages of DCE-MRI over other imaging techniques. All these features have turned DCE-MRI into the most sensitive modality for breast cancer (BC) detection and characterization as well as to assess female pelvis malignancies. Functional imaging, which allows for a more in-depth analysis of tumor vascularity, cellularity, metabolism, and oxygenation, is another possible benefit of MRI. The quantifiable metrics that these procedures generate may be employed as prognostic and response biomarkers. This would allow a timely and effective management of oncologic patients, who would be referred to the most appropriate treatment thanks to the noninvasive achievement of prognostic information.

Despite this, there are still significant challenges for imaging diagnostics and oncological patient management to develop sensitive imaging biomarkers, mainly for early therapy response prediction.

Recently, different approaches based on radiomics and artificial intelligence (AI)/machine learning (ML) applications have been proposed to overcome this knowledge gap. Radiomics is a fast-expanding field that converts digital medical pictures into quantitative data with the ultimate objective of creating imaging biomarkers as clinical practice decision support tools. The idea that radiomic data transmit information about tumor biology informs these applications. For instance, radiomic characteristics could indicate temporal and geographical variability, which is well established to be a significant factor in tumor behavior and therapeutic resistance (3). As a result, radiomics has the potential to serve as a “virtual biopsy” and, in contrast to traditional biopsies, uses noninvasive imaging that allows analysis of the entire tumor (rather than a focal sample) and can be applied more easily at multiple time points for disease monitoring, offering potentially significant diagnostic information related to disease evolution (4, 5).

In this Research Topic, four articles have been published on both ML and conventional statistics applied to DCE-MRI for the prediction of histological BC features (i.e., the discrimination between metaplastic and non-metaplastic triple negative BC) (Kong et al.) and response to chemotherapy in both breast (Li et al.) and ovarian (Lei et al.) cancers, also including the shrinkage patterns in BC (concentric vs others) (Fan et al.).

According to Lei et al., a radiomic model including DCE-MRI radiomics features from the non-manually segmented whole abdomen was more effective than that developed using radiomics features from the primary lesions to predict platinum sensitivity in 93 patients with ovarian cancer. Based on Li et al. and Fan et al.‘s experience, radiomics applied to DCE-MRI was effective in predicting the occurrence of a pathological complete response and the type of shrinkage patterns in BC (Fan et al.), respectively. In detail, ML using both pre- and early DCE-MRI examinations combined with clinical and histological information of 95 women with BC was highly effective in predicting a pathological complete response after neoadjuvant chemotherapy, obtaining an AUC of 0.925 (95% CI: 0.760–0.990) in the validation cohort (Li et al.). Similarly, the combination of multiparametric MRI panel (DCE-MRI, T2-w, DWI) and histological tumor features yielded the highest accuracy (AUC = 0.912, 95% CI: 0.802–0.985) in the prediction of tumor shrinkage pattern after neoadjuvant chemotherapy (Fan et al.). This could have strong implications to plan surgical approaches after treatment (conservative vs. radical). Finally, even a non-radiomic approach using conventional statistics proved to be feasible and effective for the noninvasive assessment of histological BC features with important prognostic information. Specifically, in a subset of 117 patients with triple-negative BC, a nomogram including tumor enhancement features on DCE-MRI, such as internal enhancement patterns and lesion type (mass vs. non-mass), was able to discriminate metaplastic and non-metaplastic triple-negative BC with an accuracy of 0.819 (95% CI: 0.693−0.946) in the training set (Kong et al.).

Despite these promising findings and the use of a training and validation cohort, which can preliminarily assess the potential generalizability of the developed models, the published studies are affected by the well-known limitations of AI and predictive studies, the first represented by the limited samples and their single-institution nature.

The lack, as with other methods, of reproducibility in multicenter contexts of the features extracted, the method’s lack of applicability in a clinical routine, and the scant evidence of a specific biological correlate not linked to a more generic, albeit important, tumor heterogeneity represent the strongest limitations to be overcome in future investigations (6).

As for AI applications in female oncological cancers, this represents a research field that is not fully explored to date.

Additional drawbacks of this approach are represented by ethical issues linked to diagnostic error responsibility, and the need for demanding and time-consuming training or manual annotations by clinical experts of a large amount of data for an efficient AI system (7).

Despite specific misgivings related to individual methods, diagnostic MRI represents a cornerstone in the workflow of the cancer patient and, with the integration of radiomics and AI, also a valuable annex towards increasingly personalized medicine.

## Author contributions

VR: Writing – original draft, Writing – review & editing. CC: Writing – original draft, Writing – review & editing.
